# On the Adulteration of the Hydriodate of Potash

**Published:** 1829-10

**Authors:** J. Pereira

**Affiliations:** Lecturer on Chemistry and Materia Medica, &c.


### HYDRIODATE OF POTASH.

*On the Adulteration of the Hydriodate of Potash.* By J. Pereira, Esq. F.L.S. Lecturer on Chemistry and Materia Medica, &c.

Having in two instances lately met with hydriodate of potash much adulterated with the carbonate of potash, and believing that this adulteration is very common, although it has not hitherto been noticed, I have taken the liberty of drawing the attention of the profession to this subject, through the medium of the London Medical and Physical Journal, and of pointing out the easiest methods of detecting it.

It is well known that iodine is very sparingly soluble in water; but that water holding in solution hydriodate of potash is capable of dissolving a larger quantity of iodine. In the General Dispensary, a solution termed Liquor Iodinæ is kept, made on this principle; that is, consisting of iodine dissolved in a solution of the hydriodate. My attention was first directed to the adulteration of this salt, by one of my assistants, who informed me that he had twice failed in making the Liquor. Thinking that he might have committed some error, I attempted to make it myself, but found that the iodine was only partially dissolved. Of course, I immediately inferred that either the iodine or the hydriodate was impure. The iodine, however, I soon found was quite pure; and I then directed my attention to the hydriodate.

The salt was observed to contain but very few crystals: those that were noticed, however, had the appearance of the hydriodate. The greater part of the salt seemed as if it had been heated, so as to destroy its crystalline form. To the taste it was powerfully alkaline, and affected very strongly vegetable colours. These characters led me to suspect that it contained an alkaline carbonate. Muriatic acid, added to a solution of it, produced effervescence: the same takes place with the pure hydriodate, owing to the separation and decomposition of the hydriodic acid; but in the case of the suspected salt, however, the gas that escaped was conducted by means of a curved tube into lime-water, which it immediately rendered milky, proving that carbonic acid was present. A solution of the suspected salt, added to lime-water, gave a white precipitate, soluble with effervescence in muriatic acid; the same coloured precipitate took place when the suspected solution was added to a solution of muriate of barytes, and effervescence was produced by the addition of muriatic acid. Sugar of lead gave a white precipitate of carbonate of lead, instead of a beautiful yellow one of iodide of lead, which the true hydriodate gives. Hence it was clear that an alkaline carbonate was present; but was it potash, or soda? To determine this I proceeded as follows.

It is well known that the salts of potash impart to flame a beautiful pale violet colour; but those of soda a pure yellow. Hence, if a salt of soda be mixed with a salt of potash, its presence may be detected by the alteration in the colour of the flame. On this principle, I determined that the substance used to adulterate the hydriodate was carbonate of potash. A piece of clean packthread was wetted with a strong solution of the suspected salt. The wetted end was then dipped into the cup of tallow immediately surrounding the wick of a candle, so that it might be enveloped in melted tallow. It was then applied to the exterior of the flame, not quite in contact with the luminous part, but so as to be immersed in the cone of invisible but intensely heated air which envelopes it. An irregular sputtering combustion of the tallow on the thread took place, and the invisible cone of heat was rendered luminous, of a *pale violet colour*. Hence, then, it did not appear that any salt of soda was present; otherwise, the colour of the flame would have been rendered more or less yellow.

Having thus satisfied myself that the impurity was carbonate of potash, I next proceeded to ascertain the quantity of it. Some of the salt was heated in a glass tube over a spirit lamp, to deprive it of water. Ten grains of the salt thus dried were dissolved in distilled water, and excess of muriate of barytes added. A precipitate, consisting of carbonate of barytes, took place, which was collected, and dried by a water bath. It weighed eleven grains. Now eleven grains of carbonate of barytes consist of

| | |
|---|---|
| Carbonic acid . . | 2,42 |
| Barytes . . . . | 8,58 |

Hence, then, there must have been 2,42 grains of carbonic acid in ten grains of the suspected salt.

Assuming, from the strong alkaline taste of the salt, its powerful effect in turning green vegetable blues, and from its precipitating sulphate of magnesia, that the carbonic acid was combined with potash in the proportion to form the Carbonate, (Subcarbonate of the Pharmacopœia,) it must, therefore, have been combined with 5,28 grains of potash. Consequently the adulterated salt consisted of

| | |
|---|---|
| Iodide of Potassium* . . . . . . . | 2,30 |
| Carbonate of Potash, (Subcarb. Ph. L.) | 7,70 |
| | 10,00 |

The quantity of iodide of potassium is here inferred from the quantity of the carbonate of potash present. That this inference is correct, there can be, I think, but little doubt: certainly, the quantity of iodide cannot have been larger than is here stated. Now, assuming that the equivalent for iodine is 125, it follows that 2,30 grains of iodide of potassium contain about 1,66 of iodine. In the following experiment I obtained 1,5 of iodine, which is a very close approximation, particularly when we consider the volatile nature of this substance.

Ten grains of the suspected salt perfectly dried, were introduced into a glass tube, and strong nitric acid added to it by means of a dropping tube. Effervescence took place, and the iodine vapour, which was evolved, condensed on the sides of the tube. The iodine was then cautiously

* When *hydriodate of potash* is heated to drive off its water, it is converted into *iodide of potassium;* but, by solution in water, the latter is converted into the *hydriodate of potash.*

sublimed into another tube inverted over the first one. To guard against moisture, the second tube, which contained the iodine, was placed under an exhausted air-pump receiver, with sulphuric acid, for a few minutes. The iodine weighed 1,5 grains. Hence, then, we have a right to conclude that the above-mentioned quantity of iodide of potassium is correct.

If, as I suspect, the adulteration of hydriodate of potash be frequently practised, it will explain why such different statements of the effects of this remedy have been made. When pure, it is a most valuable remedy in glandular and other affections. But it must be evident to every one that very different effects result from the use of subcarbonate of potash instead of the hydriodate.

I shall now make a few remarks on the best methods of detecting adulterations of this salt. The substances most likely to be met with are the Carbonates, Sulphates, and Muriates; which may be detected thus:

1. If the *carbonates* are present, they may be known by lime-water, muriate of barytes, or sulphate of magnesia, producing a white precipitate in a solution of the suspected salt, soluble with effervescence in muriatic acid. Sugar of lead also produces a white precipitate, which effervesces on the addition of muriatic acid; chloride of lead being precipitated.

I would, however, here remark, that if any of the above precipitates be small, and the quantity of fluid large, the effervescence may be hardly, or not at all perceptible, owing to the solution of the carbonic acid in the fluid.

It may happen, also, that, although the hydriodate is adulterated with the carbonate of potash, yet the white precipitate produced by muriate of barytes may not be *wholly* soluble in muriatic acid, owing to the carbonate of potash of the shops usually containing some sulphate mixed with it.

2. The *sulphates* (as of soda) may be detected by a solution of sulphate of magnesia producing no precipitate; but a solution of muriate of barytes produces a heavy white precipitate, insoluble in muriatic acid. This adulteration is, I believe, very rare.

3. The *muriates*, according to Chevallier and Robiquet, are frequently present. Indeed, the latter chemist states that they may be one of the results of the operation to form the hydriodate. The peculiar saltish taste would lead us to suspect the presence of either muriate of soda or of potash. However, we may determine this chemically.

Add a solution of nitrate of silver to the suspected solution, and a yellowish white precipitate will fall down; to which add excess of liquor ammoniæ, and stir the mixture. After letting it stand for a little time, filter. If the filtered liquid throw down a white precipitate on the addition of nitric acid, the suspected salt contained a muriate.

The theory of this process is very simple. Nitrate of silver throws down, in a solution of the pure hydriodate, a yellowish white precipitate of iodide of silver, *insoluble* in ammonia. From the solution of a muriate, the nitrate of silver throws down a white precipitate of chloride of silver, *soluble* in ammonia. Hence, then, when a solution of nitrate of silver is added to a solution of the hydriodate adulterated with a muriate, we obtain a precipitate consisting of the iodide and chloride of silver. Ammonia dissolves the chloride, but leaves the iodide. When the liquid is filtered, and an acid is added, the chloride of silver is precipitated.

The above is the only easy process for detecting the muriates that I have been able to contrive. Robiquet has published one much more complicated, and less certain in its results. It is as follows.

"Take a determinate weight of quite pure hydriodate, (suppose ten grains;) take also the same quantity of the suspected salt. Dissolve them separately in the same quantities of water; and introduce them into small tubulated retorts, to which are attached receivers. Introduce into each of these vessels, through the tubulure, excess of nitric acid, so as to decompose the hydriodate of potash: nitrate of potash will be formed, and iodine separated. By the application of a sufficient degree of heat, the whole of the iodine may be volatilized. The iodine in each vessel is to be separately dried and weighed. The difference in weight will indicate the purity of the suspected salt. Afterwards the two distilled liquids are to be tested with nitrate of silver. The liquid obtained from the pure hydriodate will not give any precipitate with the nitrate of silver; whereas, that which arises from the hydriodate adulterated with a muriate will produce a white precipitate." (*Dictionnaire des Drogues*, tome iii.; art. *Hydriodate de Potasse.*)

*General Dispensary, Aldersgate street;*

*August 14th*, 1829.

